# Challenges of Culturing Human Norovirus in Three-Dimensional Organoid Intestinal Cell Culture Models

**DOI:** 10.1371/journal.pone.0063485

**Published:** 2013-06-03

**Authors:** Efstathia Papafragkou, Joanne Hewitt, Geun Woo Park, Gail Greening, Jan Vinjé

**Affiliations:** 1 Division of Viral Diseases, Center for Disease Control and Prevention, Atlanta, Georgia United States of America; 2 Center for Food Safety and Applied Nutrition, Division of Molecular Biology, Food and Drug Administration, Laurel, Maryland, United States of America; 3 Institute of Environmental Science and Research Ltd, Kenepuru Science Centre, Porirua, New Zealand; Columbia University, United States of America

## Abstract

Human noroviruses are the most common cause of acute gastroenteritis worldwide. Recently, cell culture systems have been described using either human embryonic intestinal epithelial cells (Int-407) or human epithelial colorectal adenocarcinoma cells (Caco-2) growing on collagen-I porous micro carrier beads in a rotating bioreactor under conditions of physiological fluid shear. Here, we describe the efforts from two independent laboratories to implement this three dimensional (3D) cell culture system for the replication of norovirus. Int-407 and Caco-2 were grown in a rotating bioreactor for up to 28 days. Prior to infection, cells were screened for the presence of microvilli by electron microscopy and stained for junction proteins (zonula occludens-1, claudin-1, and β-catenin). Differentiated 3D cells were transferred to 24-well plates and infected with bacteria-free filtrates of various norovirus genotypes (GI.1, GI.3, GI.8, GII.2, GII.4, GII.7, and GII.8). At 12 h, 24 h, and 48 h post inoculation, viral RNA from both cells and supernatants were collected and analyzed for norovirus RNA by real-time reverse transcription PCR. Despite observations of high expression of junction proteins and microvilli development in stained thin sections, our data suggest no significant increase in viral titer based on norovirus RNA copy number during the first 48 h after inoculation for the different samples and virus culture conditions tested. Our combined efforts demonstrate that 3D cell culture models using Int-407 or Caco-2 cells do not support norovirus replication and highlight the complexity and difficulty of developing a reproducible *in vitro* cell culture system for human norovirus.

## Introduction

Human noroviruses are recognized as the most common cause of outbreaks of acute gastroenteritis worldwide [1]. On the basis of similarity in amino acid sequences of the major capsid protein, human norovirus are classified into 5 genogroups (GI-GV), and each genogroup is further divided into genotypes [2]. The majority of the outbreaks are caused by GII.4. Since the detection of Norwalk virus, the prototype strain for norovirus, in stool samples of patients with acute gastroenteritis in 1968, many investigators have attempted to develop a cell culture system to better study the virus and assess the effectiveness of control measures such as disinfectants [3]. Duizer and colleagues tested a comprehensive number of human and animal cell lines including primary kidney cell lines, primary intestinal cell lines and colon carcinoma cell lines, none of which was able to support replication of human norovirus [4]. While murine norovirus, which was discovered in 2003, can be cultured successfully in dendritic and macrophage cell lines [5], [6], those cell types are unable to support propagation of human norovirus [7]. More recently, freshly collected adult human duodenal tissues were infected successfully with a GII.4 strain as demonstrated by virus RNA production and immunohistochemical staining; however, when a human intestinal epithelial cell line from fetal ileum tissue was infected, only limited virus replication was detected [8]. More recent findings suggested that human norovirus may have a tropism for non-epithelial cells of the human duodenum [9].

The use of a rotating wall vessel (RWV) bioreactor was first reported by the National Aeronautics and Space Agency [10]. These RWV bioreactors are capable of simulating microgravity which can make cells more permissible to viral infection and replication [11]. Cells growing in a RWV system form three-dimensional (3D) cellular aggregates and by using such 3D cell models higher yields of rhinovirus and [12] and hepatitis C virus have been reported [13]. Recently, a 3D organoid model generated in a RWV system was reported to successfully support human norovirus replication in a human embryonic small intestinal epithelial cell line (Int-407) [14] as well as in a human epithelial colon rectal adenocarcinoma cell line (Caco-2) [15]. To replicate these findings, we initiated a collaborative study by the Centers for Disease Control and Prevention in the United States (Lab A) and the Institute of Environmental Science and Research in New Zealand (Lab B). The aim of our investigation was to implement the 3D culture methodology for norovirus, test a larger number of norovirus strains, and evaluate several different culture conditions to assess the robustness of the system.

## Materials and Methods

### Cell lines

Int-407 (CCL-6) and Caco-2 (HTB-37) cells were originally obtained from the American Type Culture Collection (ATCC, Manassas, VA). In addition, in one experiment performed by Lab A, Int-407 cells cultured in Dr. Cheryl Nickerson's laboratory (The Biodesign Institute, Arizona State University) and Int-407 cells cultured in Lab A were inoculated with the same virus side by side. Cells were grown as standard monolayers in GTSF-2 medium (Hyclone, Logan, UT) containing 100 U/ml penicillin, 100 µg/ml streptomycin and 0.5 µg/ml Fungizone® (Invitrogen, Carlsbad, CA) at 37°C and 5% CO_2_ as described [14]. GTSF-2 medium is a specially designed triple-sugar based minimal essential medium supplemented with 10% heat-inactivated fetal bovine serum (Lab A, Hyclone, Pittsburg, PA; Lab B, Invitrogen), 26.2 mM NaHCO_3_, and 2.5 mg/l insulin-transferrin-sodium selenite solution [16], and used by the Straub team [14]. When the cells were approximately 80% confluent, they were washed with pre-warmed Hanks Balanced Salt Solution (Invitrogen) in Lab A or 1×10 mM phosphate buffered saline pH 7.2 (PBS) in Lab B, trypsinized and added into the 3D vessels following the procedure described below.

### Three-dimensional cultures (Int-407 and Caco-2)

Three hundred micrograms of hydrated sterile pre-washed Cytodex®-3 microcarrier beads (Sigma, St. Louis, MO), which are collagen type-I-coated porous microspheres of an average 175 µm diameter were seeded into a Slow Turning Lateral Vessel (STLV) (Synthecon, Houston, TX) with approximately 2×10^6^ cells (Int-407 or Caco-2) in a total volume of 50 ml of complete GTSF-2 medium. The cells were incubated for 30 min to allow them to bind to the Cytodex®-3 beads, after which the rotating speed of the vessel was set at 18–20 RPM to keep the cell-bead aggregates in suspension. During the first 10 days of culture, 90% of the total vessel volume was replenished by fresh complete GTSF-2 medium every 48 h, and then on a daily basis. After 18–21 days, the cells were transferred from the STLV to a High Aspect Rotating Vessel (HARV) (Synthecon, Inc, Houston, TX) to promote development of large cell aggregates and cultured for another seven days. In Lab-A, Caco-2 cells were cultured in parallel in standard monolayers under the same growth conditions.

### Human norovirus strains

Human norovirus positive stool samples of different genotypes (Table 1) were prepared as 10% clarified fecal suspensions in PBS pH 7.2, and filtered through a 0.22 µm filter (Lab A) or clarified by low-speed centrifugation (Lab B). The viral RNA copy numbers of the samples were determined by real-time RT-qPCR [17]. In Lab A, the samples were also tested for the presence of enteroviruses by cell culture using BGM cells [18] and by real-time RT-qPCR [19].

**Table 1:** Information on the 19 norovirus strains used in the study.

Strain	Trial	Genotype/year	Cell Line	Lab	Sample Identi fication
1	1	GII.4/2008	Int-407	A	2008729673
2	1	GII.4/2007	Int-407	A	2007744141
3	1	GII.7/2007	Int-407	A	2007745595
4*	2	GII.4/2007	Int-407	A	2007744141*
5	2	GII.4/2007	Int-407	A	2007745596
6	1	GII.4/2008	Caco-2	A	2008729893
7	3	GI.4/2008	Caco-2	A	2008729945
8	1	GII.4/2007	Int-407	B	20070803
9	1	GII.2/2007	Int-407	B	20070320
10	2	GI.1/1968	Int-407	B	23–3**
11	2	GI.8/2007	Int-407	B	20070922
12	2	GII.2/2007	Int-407	B	20070320
13	2	GII.4/2007	Int-407	B	20070334
14	2	GII.4/2007	Int-407	B	20071002
15	2	GII.8/2007	Int-407	B	20070399
16	3	GI.3/2008	Int-407	B	20080095
17	3	GII.4/2007	Int-407	B	20070412
18	1	GI.3/2008	Caco-2	B	20080095
19	1	GII.4/2007	Caco-2	B	20070412

All strains were used as sole infections unless otherwise noted.

* strain was the first passage of trial 1.

** Norwalk virus (GI.1) was a kind gift from Dr Christine Moe.

### Immunofluorescence and electron microscopy

The development and localization of tight junction proteins, including β-catenin and claudin, was monitored weekly for both cell lines as follows. Each week, an aliquot of cells was removed from the bioreactors, rinsed with PBS, and fixed with 4% paraformaldehyde for 25 min at room temperature. PBS was used for all subsequent washes. After three washes, cells were incubated for 10 min with 50 mM NH_4_Cl to quench free aldehydes, immediately washed again with PBS, and incubated with 0.2% Triton X-100 for 15 min. After washing, the cells were washed and blocked with either 8% heat-inactivated goat serum (Lab A) or 8% BSA (Lab B) in PBS containing 0.05% Triton-X 100 for 2 h at room temperature. After a brief wash step, aliquots of cells were incubated overnight at 4°C with antibodies specific for zonula occludens-1 (ZO-1), claudin-1, and β-catenin according the manufacturers' instructions (Zymed Laboratories, Invitrogen). After three washes, cells were counterstained with 4,6-di-amidino-2-phenylindole (DAPI) (Invitrogen) and further incubated in the dark for 1–2 h at room temperature with either Alexa-Fluor 488 labeled anti-donkey IgG antibodies (Lab A), or Alexa-Fluor 488 anti-mouse IgG antibody (Lab B) for ZO-1 and β-catenin, and Alexa-Fluor 568 labeled goat anti-rabbit antibody for Claudin-1 expression in both labs. All antibodies were purchased from Invitrogen. Cells with mounted onto glass slides with Prolong-Antifade medium (Invitrogen) and were analyzed using a confocal laser scanning microscope (Zeiss Axioplan II, Carl Zeiss, Thornwood, NY).

For visualization of microvilli in Caco-2 cells, cells were fixed in 2.5% glutaraldehyde, and agar-immobilized with 2% agar and and further processed as described previously [20]. Briefly, following post fixing and subsequent staining with 1% uranyl acetate, the cells were impregnated with 100% epoxy resin (Ernst F. Fullam, Inc., Latham, NY) and ultrathin sections were viewed using transmission electron microscopy.

### Norovirus inoculation of 3D aggregates

After 7 days in the HARV, cell aggregates were removed, washed in serum-free GTSF-2 medium, and 24-well plates were seeded with 1 ml of 10^6^ cells per well using a wide bore pipette tip. Int-407 or CaCo-2 cell aggregates were allowed to settle in the plates for about 10 min before medium was carefully removed to a final volume of approximately 0.3 ml. Diluted norovirus samples (0.1 ml) were added to duplicate wells of cell aggregates. Serum-free GTSF-2 medium was used as uninfected control. The aggregates were incubated for 1 h at 37°C and 5% CO_2_ after which serum-free GTSF-2 medium was added to 1 ml. In addition, Lab B also inoculated a mixture of norovirus GI.3 and GII.4 directly into a HARV containing Int-407 aggregates. Cells and culture medium were collected at 1 h, 12 h, 24 h, and 48 h post inoculation (pi). For the Caco-2 experiments performed in Lab B, cells and culture supernatant were collected separately. All samples were stored at −80°C until further testing.

### RNA isolation and quantitative real-time RT-qPCR

Viral RNA was extracted using 4M guanidine isothiocyanate-based lysis buffer using silica spin column (Omega Bio-Tek, Norcross, GA) [21] (Lab A), or using the High Pure Viral Nucleic Acid Kit (Roche, Mannheim, Germany) (Lab B with Int-407 cells, Caco-2 cells, experiment 1) or using the Corbett CAS1820 X-tractor Gene (Corbett Life Science, Sydney, Australia) (Lab B, Caco-2 cells, experiment 2).

Real-time RT-qPCR was carried out as described previously [22] using the QuantiTect® Probe RT-PCR kit (Qiagen, Germantown, MD) on a ABI 7500 Realtime PCR platform (Applied Biosystems, Forster City, CA) (Lab A) or the Platinum Quantitative RT-PCR Superscript III One step System (Invitrogen) on a RotorGene analyzer (Corbett Life Science) (Lab B). For Lab A, RT was carried out for 30 min at 50°C, followed activation of the Taq polymerase for 15 min at 95°C, and 45 cycles of 95°C for 10 sec, 55°C for 30 sec, and 72°C for 15 sec. In Lab B, RT was performed for 15 min at 50°C, followed by incubation for 5 min at 95°C, and then 45 cycles of 95°C for 15 sec, and 56°C for 60 sec. In each experiment a standard curve of triplicate 10-fold serial diluted GI.4 or GII.4 RNA run-off transcripts (Lab A) [23]or purified DNA plasmids generated from GI.1 and GII.3 (Lab B) were included to calculate RNA copies/ml [24].

## Results

### Three dimensional aggregates and tight junction markers

**Figure 1:**
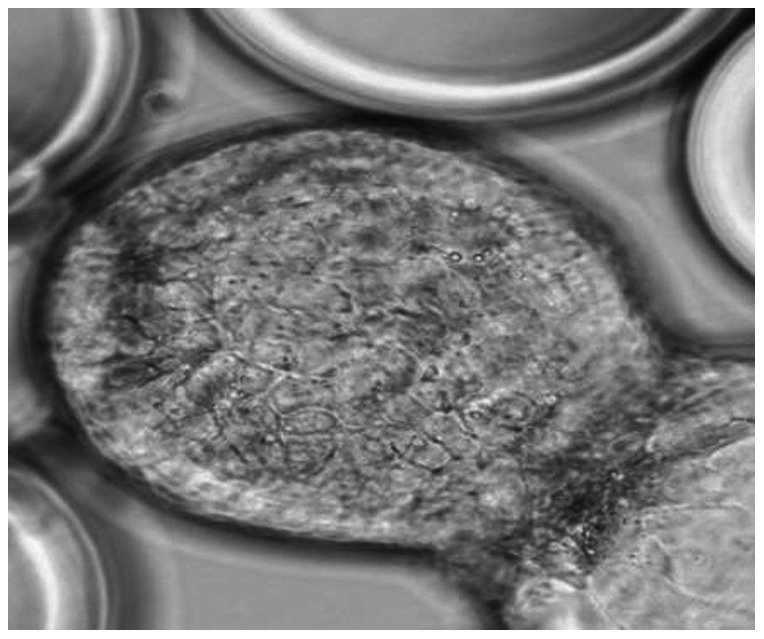
Formation of a uniform monolayer around the collagen beads coated with Int-407 cells. Cells were removed from an STLV after 7 days of culture in GTSF-2 medium at 37°C and the 3D structures visualized by light microscopy (1∶40 magnification).

**Figure 2:**
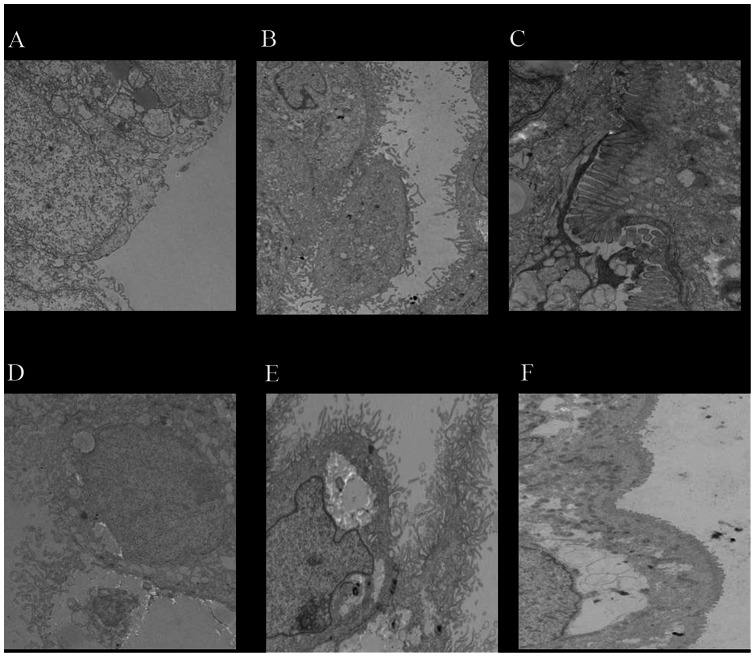
Electron micrographs of microvilli development in Caco-2 cells during a 5-week growth period. Caco-2 cells were cultured in GTSF-2 medium for up to 5 weeks at 37°C. At 2, 3 and 5 weeks of culture, under either 2D, or 3D conditions in a rotating wall vessel, cells were removed and processed for visualization under electron microscope. Top Panel [A (1200x), B (440x), C (1400x)]: 3D structures Lower Panel [D (690x), E (890x), F (690x)]: 2D structures.

**Figure 3:**
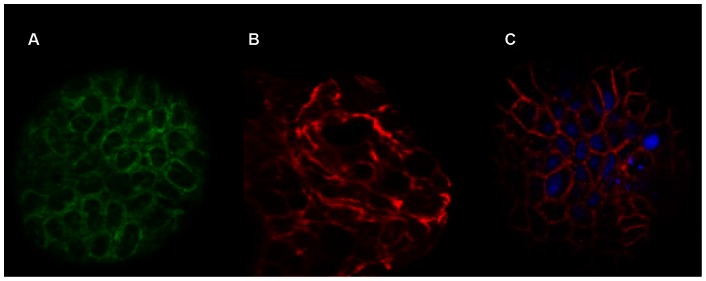
Immunohistochemical imaging of differentiation markers in Int-407 and Caco-2 cells. Image A. β-catenin localization in three-dimensional structures of Caco-2 cells after 3 weeks in culture in STLV (1∶40). Image B. Localization of ZO-1 in 3D of Int-407 cells after 4 weeks of culture in STLV and HARV (1∶40). Image C. Distribution of claudin in 3D of Caco-2 cells after 3 weeks in culture in STLV (cell nuclei were stained with DAPI, 1∶40 magnification).

Typical yields of 3D cells from the HARV ranged from 1–3×10^6^ cells/ml for both Lab A and B. Formation of cell aggregates on the collagen beads was monitored for every experiment (Figure 1), and cells were transferred to 24-well plates. Differentiation of Caco-2 cells in the 3D system start developing within the first two weeks of initial seeding in the STLV (Figure 2) and subsequently progressed so that at the time of norovirus infection of the Caco-2 cells, the microvilli were numerous and well-formed and stayed intact during the infection experiments (data not shown). Cell viability was determined on a weekly basis and was consistently found to be at least >90% (data not shown). High levels of tight junction proteins including β-catenin and claudin were detected in the 3D aggregates (Int-407 or Caco-2) (Figure 3).

### Norovirus infection

**Figure 4:**
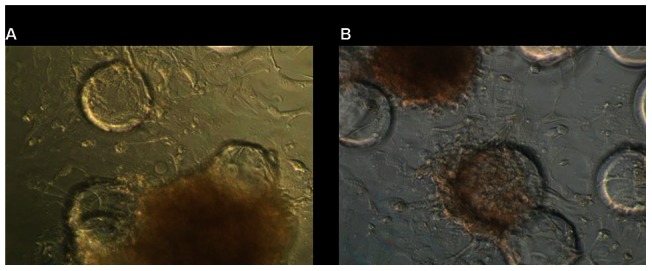
Morphology of 3D Int-407 cells after inoculation with norovirus. Images A and B: control Int-407 cells (left) and (right) Int-407 cells inoculated with GII.4 norovirus at 48 h post inoculation (light microscopy, 1∶20 magnification).

**Figure 5:**
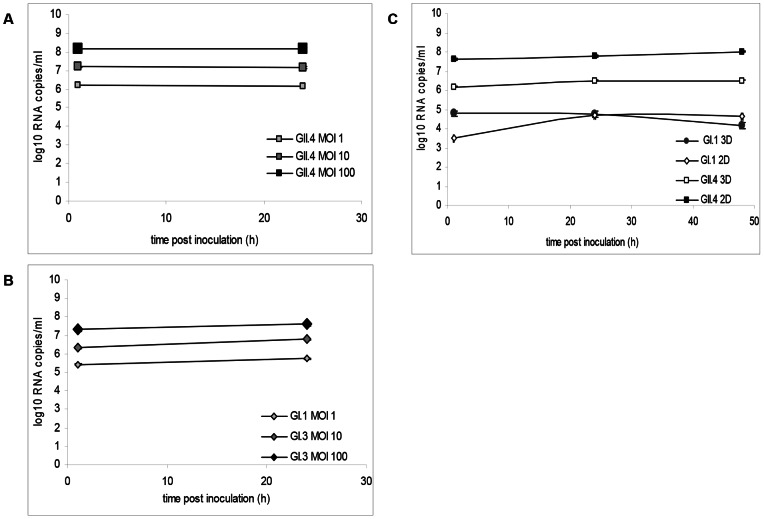
No evidence of Norovirus replication in 3D Caco-2 cells. No significant increase of RNA copy number was detected in supernatants of Caco-2 cells harvested at 0, 24 and 48 hours post inoculation. Different norovirus strains and different virus concentrations were evaluated. Viral RNA was detected by quantitative reverse transcription-realtime RT-PCR. Inoculation of 3 different concentrations (MOI 1, 10, 100) of: A) GII.4 (#19 in table 1) on 3D Caco-2 cultures (Lab B) and B) GI.3 strain (#18 in table 1) on 3D Caco-2 cultures (Lab B). C) Comparison of 2D and 3D Caco-2 cells by GI.3 (#7 in table 1) and GII.4 (#6 in table 1) (Lab A).

In all conditions tested during infection (infection medium composition, incubation period, static or rotating culture conditions, different norovirus strains), the 3D cells remained attached to the beads and viable throughout the experiments. The Int-407 or Caco-2 cell 3D aggregates showed no morphological changes or apparent cytopathic effect (CPE) compared to the control cells following 48 h of infection (Figure 4). None of the Int-407 or Caco-2 cell culture supernatants collected at 12 h, 24 h, and 48 h after infection showed an increase in the viral RNA titer compared with 1h post inoculation (Table 2 and Figure 5). In addition, none of the additional infection trials including a blind-passage (GII.4 sample (# 4 in Table 1), increasing the virus titer of the inoculum (Table 2), infecting with a mixture of norovirus strains (GI.3/2008 and a GII.4/2007) (Table 2), or infection of Int-407 cells directly in the HARV, resulted in an increase in viral RNA titer.

**Table 2:** No evidence of Norovirus replication in 3D Int-407 cells.

	Sample ID	MOI	Time post-infection (hr)			
			1	12	24	48
Lab A #1	GII.4	1	3.76±0.44	3.66±0.05	3.33±0.02	3.60±0.17
Lab A #1*	GII.4	1	3.32±0.02	3.47±0.10	3.15±0.57	3.63±0.12
Lab A #1	GII.4.	0.1	2.61±0.28	2.57±0.05	3.04±0.43	2.59±0.07
Lab A #2	GII.4	0.5	1.70±0.47	1.34±0.01	1.98±0.63	1.82±0.65
Lab A #3	GII.7	5	6.04±0.09	6.34±0.07	5.96±0.18	6.36±0.07
Lab A #4	GII.4	1	4.73±0.03	3.12±0.30	3.08±0.94	3.02±0.62
Lab A #5	GII.4	1	3.72±0.06	3.38±0.05	3.44±0.05	3.27±0.15
Lab B #8	GII.4	0.1	4.97±0.24	nd	4.79±0.09	5.04±0.14
Lab B #9	GII.2	0.1	5.16±0.18	nd	5.03±0.11	5.33±0.07
Lab B #10	GI.1	0.1	4.99±0.35	nd	4.99±0.03	4.65±0.32
Lab B #11	GI.8	1	5.92±0.14	nd	5.92±0.04	5.73±0.19
Lab B #12	GII.2	1	6.51±0.05	nd	6.29±0.09	6.19±0.11
Lab B #13	GII.4	0.001	3.26±0.08	nd	3.19±0.10	2.42±0.29
Lab B #14	GII.4	10	7.51±0.07	nd	6.97±0.02	6.71±0.26
Lab B #15	GII.8	0.1	5.12±0.08	nd	5.08±0.14	4.46±0.24
Lab B #16	GI.3	0.01	4.19±0.07	nd	nd	4.12±0.07
Lab B #16**	GI.3	0.01	4.16±0.08	nd	nd	4.27±0.07
Lab B #17	GII.4	0.01	3.68±0.09	nd	nd	3.36±0.07
Lab B #17**	GII.4	0.01	3.56±0.06	nd	nd	3.50±0.11

The RT-qPCR data are expressed as the average log RNA copies/ml of at least two replicates ± standard deviation.

* cells were obtained from Arizona State University; ** indicates a mixed infection; nd: not determined.

When the Caco-2 cell monolayers were inoculated with a mix of GI and GII strains (Lab A), no increase of viral RNA could be detected after two days incubation (Figure 5C). Similarly, when 3D aggregates of Caco-2 cells were inoculated with a variety of norovirus strains, there was no significant increase in the viral RNA at 24 h (Figure 5A, B, Lab B) or 48 h (Figure 5C, Lab A) post infection. To confirm that the viral RNA that was detected 48 h after infection most likely represented the virus inoculum, we tested whether the virus was stable in infection medium at 37°C for 48 h and found no decrease of viral RNA titer (Lab B, data not shown). In several infection experiments the cell culture medium and cells were separated and analyzed separately (Lab B), but no change in virus titer was found.

## Discussion

The development of a reliable cell culture system for human norovirus is essential to better understand basic biology of the virus and to better assess the effectiveness of control measures to limit norovirus outbreaks. The purpose of this study was to replicate the recent publications on the successful replication of human norovirus in 3D aggregates of Int-407 and Caco-2 cells [14], [15].

We used protocols that were similar to the published procedures including a specially formulated GTSF-2 medium [16]. Cells were added to collagen-coated beads and grown under microgravity conditions into 3D aggregates. Several reports have described the use of collagen-coated microcarrier beads [12] and 3D cell culture systems for increased virus yields [13] although not in all cases [25].

Int-407 cells grown under 3D culture conditions develop tight junctions, produce mucus and have differentiation characteristics normally found in tissues *in vivo* [26]. We confirmed the increased expression of tight junction proteins indicating that the cells used in our experiments were highly differentiated. Since cell lines in different laboratories may develop slightly different characteristics due to the use of different culture conditions, we infected a batch of Int-407 cells from Arizona State University that had been used in the experiments described by Straub in parallel with a batch of cells maintained in Lab A with a GII.4 positive stool, but no increase in viral RNA titer 48 h post infection was observed. When we infected 3D Int-407 cells with a mixture of human norovirus strains from both GI and GII viruses, no increase in viral RNA titer was detected 48 h post infection.

Caco-2 cells have been used extensively as a candidate cell line for human norovirus after the report that Norwalk virus-like particles could bind and internalize to differentiated Caco-2 cells [27]. In addition, these cells have been used successfully for culture of a variety of enteric viruses including rotaviruses, poliovirus, coxsackieviruses, and astroviruses [28]. Caco-2 cells progressively developed typical brush border microvilli which were more organized and dense in 3D complexes compared to standard (2D) monolayers suggesting a higher differentiation level of the 3D aggregates. A four-week period was considered adequate for the cells to grow in the 3D vessels as prolonged incubation resulted in cells stacking on top of each other, thus destroying the desired cell formation on the beads resembling the normal intestinal epithelium *in vivo* [26]. For detection of norovirus replication in cells we used quantitative real time RT-qPCR. We did not detect any increase in norovirus titer following infection of Int-407 or Caco-2 cells by real time RT-qPCR. Fluorescent in situ hybridization with specific norovirus molecular beacon probes [14] was trialed for norovirus detection in cells but autofluorescence was a problem and so this method was not further pursued.

Our findings of absence of increase in viral RNA titer in conjunction with the presence of intact microvilli when the cells were infected is in agreement with reports of norovirus infections *in vivo* where blunted villi and partially shortened microvilli could be seen on intact epithelial cells in experimentally infected human volunteers [29] and naturally infected norovirus patients [8].

Human volunteer studies have shown that subjects with mutations in their fucosyltransferase (FUT2) gene are resistant to Norwalk virus infection [30], [31]. Without a functional FUT2 gene certain histoblood group antigens (HBGA) cannot be generated and binding studies using virus-like particles have shown that Norwalk virus binds to certain HBGA [32]. However, different norovirus genotypes recognize different HGBAs and up to eight receptor-binding patterns have been described [33].

Differentiated Caco-2 cells have been shown to express high levels of H-type 1 carbohydrates [34] as well as type 3 and 4 carbohydrates [27] but as we did not perform HBGA phenotyping on Caco-2 or Int-407 cells, we were unable to confirm if H-type or Lewis carbohydrates were expressed on the surface of these cells. A recent study reported the lack of expression of H type 1 and 2 or Lewis b antigens on differentiated 3D cultures of Int-407 cells [35] which may explain why the cells used in our study are not suitable to study norovirus replication. However, even overexpressing of the human FUT2 gene is not sufficient to allow complete norovirus replication [36].

Apart from the permissibility of a particular cell line or *in vitro* growth conditions, several other factors may explain the negative culture results including the infectiousness of the norovirus strains used. All norovirus strains had been stored at −80°C which is similar to conditions used to challenge human volunteers [37].

No CPE-like morphological changes or cytotoxicity was observed in any of the infection trials on 3D Int-407 or 3D Caco-2 cells. Our findings are in contrast with observations by Straub [14] who reported CPE-like morphological changes of the 3D aggregates after inoculation with different norovirus strains. However, their CPE may not have been norovirus specific as has been previously described [4] and a recent report suggested that the observed CPE may have been caused by lipopolysaccharides or other stool contaminating toxins [35].

Our study has several limitations. First, we did not test the 3D cell aggregates for the presence of HBGA on the surface of the cells which has been shown to be critical for the initial binding of norovirus to cells [32]. Second, we did not perform multiple blind passages to try to adapt the viruses to the cells. Many attempts have been made to develop a reproducible cell culture system for human norovirus but none of them have been successful [4], [7], [35]. Using either Int-407 or Caco-2 cells grown under microgravity conditions as reported previously [14], [15], we found no evidence of norovirus replication using these models. Transfecting cells with Norwalk virus RNA resulted in successful virus replication and protein synthesis, which suggests that the likely block in replication occurs after binding of the virus to the cell and prior to viral entry [36]. Recently, human intestinal organoids derived from pluripotent stem cells have been reported as a promising new model for generating intestine-like tissue [38] and successful replication of rotaviruses in these organoids not only in epithelial cells but also in the mesenchymal cell population [39] supports the hypothesis that cells other than those of epithelial origin should be employed to test whether they support successful norovirus replication *in vitro* [9].
